# Acupuncture promotes white adipose tissue browning by inducing UCP1 expression on DIO mice

**DOI:** 10.1186/1472-6882-14-501

**Published:** 2014-12-16

**Authors:** Weixing Shen, Yang Wang, Sheng-Feng Lu, Hao Hong, Shuping Fu, Suyun He, Qian Li, Jingxin Yue, Bin Xu, Bing-Mei Zhu

**Affiliations:** Key Laboratory of Acupuncture and Medicine Research of Ministry of Education, Nanjing University of Chinese Medicine, 210023 Nanjing, China; School of Information Technology, Nanjing University of Chinese Medicine, 210023 Nanjing, China

**Keywords:** Acupuncture, Browning, Obesity, UCP1, White adipose tissue

## Abstract

**Background:**

To study the influence of acupuncture and its possible mechanism on white adipose tissue of high fat diet-induced obese.

**Methods:**

Four-week-old C57BL/6 J mice were randomly divided into a normal diet group and a high-fat diet (HFD) group. After 8 weeks, the HFD mice were randomly divided into Electro-acupuncture (EA) group and control group. Mice in the EA group were electro-acupunctured, under physical restraint, on Zusanli (ST36) and Neiting (ST44) acupoints, while the mice in the control group were under physical restraint only. Body weight and food intake were monitored, and serum leptin, cholesterol and triglyceride levels were measured by using biochemistrical methods. The effect of EA on white adipose tissues (WAT) was assessed by qPCR, immunobloting, immunohistochemistry (IHC), immunoprecipitation and cold endurance experiment.

**Results:**

The WAT/body weight ratio decreased (*P* < 0.05) in the EA group, albeit no significant difference on food consumption between EA and control groups. The difference in the darkness of Epi-WAT between EA and control groups could be distinguished visually. HE staining indicated that the EA mice had an increased number of UCP1-immunoreactive paucilocular adipocytes in their WAT. The expressions of brown adipose tissue (BAT) markers, including UCP1, COX4il and Nrtf1 were increased in the WAT of EA mice, acetylation of Pparγ was decreased by electro-acupuncture.

**Conclusion:**

EA can remodel WAT to BAT through inducing UCP1 expression, and this may be one of the mechanisms by which acupuncture affects weight loss.

## Background

Obesity is one of the leading global health risk factors and is associated with other health concerns, such as type 2 diabetes mellitus, cardiovascular diseases, and cancer. In high-risk subjects, a 5-10% reduction in body weight can lower the risk of diabetes by 58% [1]. Existing anti-obesity drugs and therapies have been limited by side effects including mood changes, suicidal thoughts, and gastrointestinal or cardiovascular complications, and have also failed to achieve adequate weight control in all patients [2]. In this way, obesity has become a modern-day medical challenge.

Obesity results from chronic excess energy intake over energy expenditure. Its progression is controlled by variable and complex interactions between genetic background, environmental factors, behavioral factors, and socioeconomic status. In periods of excess caloric intake, energy is accumulated mainly in the white adipocytes of white adipose tissue (WAT), in the form of triacylglycerols that can be mobilized when needed, releasing fatty acids into the blood stream. Brown adipose tissue (BAT) is different from WAT at a functional, morphological, and molecular level. BAT dissipates energy directly as heat by uncoupling fatty acid oxidation from ATP production via uncoupling protein-1(UCP1), which is characteristically expressed in the mitochondrial inner membrane of brown adipocytes and, when activated, uncouples the oxidation of fuel substrates from the production of ATP, thus generating heat [3]. UCP1 is also vital for the regulation of body temperature [4], and is involved in the control of body weight [5]. Recently, inducible brown-like adipocytes from white adipose tissue, in response to appropriate stimulation, have become the target of many obesity therapeutics [6–9]. Novel approaches using specific drugs or other stimulations to induce a program of brown fat differentiation, including UCP1 expression and enhanced oxidative metabolism, in white adipose cells are expected to be on the rise.

For many years, acupuncture has been used to treat obesity in China and has been studied in clinical trial around the world [10–12]. It provides a safe and effective alternative therapy, gaining increased acceptance by obesity patients as their treatment of choice, as well as recognition by both the National Institutes of Health and the WHO. However, the mechanism by which acupuncture targets obesity still remains unclear.

Does electro-acupuncture (EA) stimulation induce WAT “browning”? In the present study, we employed diet induced obese mice as the animal model, and treated them with EA on Zusanli (ST36) and Neiting (ST44) acupoints. Our study, for the first time, revealed that electro-acupuncture treatment could induce UCP1 expression in WAT and promote browning of WAT.

## Methods

### Antibodies and primers

#### Antibodies

Goatanti-UCP1 (Santa Cruz, Cat#sc-6528, 1:500 dilution for Western Blot); Rabbit anti-Prdm16 (Abcam, Cat#ab106410, 1:1000 dilution for Western Blot); Rabbit anti-SirT1 (Abcam, Cat#ab32441, 1:1000 dilution for Western Blot); Rabbit anti-GAPDH (Cell Signaling, Cat#2118, 1:2000 dilution for Western Blot); Rabbit anti-Pparγ for western blot (Cell Signaling, Cat#2443, 1:1000 dilution for Western Blot); Mouse anti-Pparγ for immunoprecipitation (Santa Cruz, Cat#sc-7273); Mose anti-Acetyl-Lycine (Cell Signaling, Cat#05-515, 1:1000 dilution for Western Blot).

#### Primers

Primers were designed using Primer 5.0 software. UCP1 (forward: 5′-TGGAAAGGGACGACCCCTAA-3′, Reverse: 5′-CAGGAGTGTGGTGCAAAACC-3′), Prdm16 (Forward: 5′-CTTAGCCGGGAAGTCACAGG-3′, Reverse: 5′-CCTCAACACACCTCCGGGTA-3′), Pparγ (Forward: 5′-GTCACACTCTGACAGGAGCC-3′, Reverse: 5′-CACCGCTTCTTTCAAATCTTGT-3′), GAPDH (Forward: 5′-AAGGGCTCATGACCACAGTC-3′, Reverse: 5′-CAGGGATGATGTTCTGGGCA-3′).

#### Animals and grouping

Four-week-old C57BL/6J mice (n = 100) were supplied by the Experimental Animal Center of Nanjing University of Chinese Medicine, and were randomly divided into normal diet group (NF, n = 20) and high-diet food group (HDF, n = 80). Mice in NF group were fed a normal diet, and mice in HDF group were fed D12451 Rodent Diet with 45 kcal% Fat (purchased from Shanghai SLAC Laboratory animal Co. Ltd). Mice were weighted each week after fasting for eight hours. After eight weeks, obese mice were defined by a 20% increase in body weight compared to the NF mice, and were then randomly divided into control group and Electro-acupuncture group (EA). The study was approved by the Institutional Animal Care and Use Committee of Nanjing University of Chinese Medicine, and all procedures were conducted in accordance with the guidelines of the National Institutes of Health Animal Care and Use Committee.

#### Electro-Acupuncture intervention

Mice in the EA group were physically restrained, then electro-acupunctured on Zusanli (ST36) and Neiting (ST44), while mice in the control group were restrained in the same way, without acupuncture. For the EA mice, two acupuncture needles (Gauge-28, 0.5 cm) were separately inserted into each acupoint and an electrical current was provided to the needles through an electrical stimulator with parameters of 2/15 Hz at an intensity level of 1 mA (Han Acuten, WQ1002F, Beijing, China) for 30 minutes, once a day, six days a week. Mice body weight and food consumption were monitored every week. After 4 weeks of EA treatment, all of mice were sacrificed with CO_2_ and samples were collected.

#### qPCR analysis

Total RNA was isolated using TRIzol® Reagent (Invitrogen, Cat#15596-026) according to the manufacturer’s recommendations. RNA concentrations were quantified and reverse-transcribed using ThermoScript™ RT-PCR System for First-Strand cDNA Synthesis (Invitrogen, Cat#11146-016). Gene expressions were detected using GoTaq qPCR Master Mix (Promega, Cat#A6001) in Strata gene MX3000P Real-Time PCR system (Genetimes, China). Relative gene expression levels were calculated by △△Ct and compared with GAPDH as internal control.

#### Immunoblotting

Samples in SDS Loading Buffer were heated (100°C, 5 min), subjected to SDS-PAGE, transferred to PVDF or nitrocellulose membranes, and blocked (4°C, overnight) in PBST (PBS with 0.05% Tween 20) containing 5% non-fat dry milk or 5% BSA. Blots were incubated with a primary antibody in blocking buffer (overnight, 4°C), and then with a second antibody (1:1000 ~ 2000 dilution, 1 h, RT). Signals were detected using SuperSignal® West Femto Maximum Sensitivity Substrate. Immunodetection of endogenous GAPDH was utilized to indicate that equivalent amounts of protein were present in samples added to the SDS PAGE (wells/lanes.μg/L).

#### Immunoprecipitation

Rat epididymal adipose tissue were homogenized in ice-cold buffer containing 10% glycerol with mmol/L of 50 HEPES, 100 sodium pyrophosphate, 100 sodium fluoride, 1 EDTA, 1X Protease inhibitor cocktail (Sigma, Cat# P8340) and 1% Triton X-100 or 1% NP-40. Insoluble material was removed by centrifugation (100,000 g, 30 min, 4°C). Pre-cleared lysates (500 μg) were incubated (overnight, 4°C) with 2 μg anti-Pparγ (Santa Cruz). Protein G beads were added; After incubated for 2 hours at 4°C, the beads were centrifuged (2,000 g, 30 s), washed 5 times with ice-cold lysis buffer, and heated in 1× SDS sample buffer containing 5% β-mercaptoethanol at 95°C for 5 minutes to release bound protein. Eluates were subjected to SDS-PAGE for Western blot.

#### Elisa detection of serum leptin, cholesterol and triglyceride level

Elisa kit were purchased from ShangHaiQiaDu Biotechnology CO.LTD, Leptin (Cat# QD20613), cholesterol (Cat#QD20142) and Triglyceride (Cat# QD20195). Serum level were detected according to the manufacturer’s recommendations. Briefly, standards and each 40 μl of mice serum was added into 96 well coated plate with 10 μl biotin labeled antibody, incubated at 37°C for 30 min and followed with 5 times washing, then incubated with HRP-Conjugate reagent for 60 min at 37°C. Washed 5 times, then add Chromogen solution A 50 μl and Chromogen solution B, evade the light preservation for 15 min at 37°C, 50 μl stop solution were added, OD values were detected at 450 nm by using Multiskan FC (Thermo scientific, USA). Leptin, cholesterol and triglyceride level were quantified by standard curve.

#### Histological analysis and Immunofluorescence staining

Adipose tissue was fixed with 4% formalin and embedded with paraffin, sliced to 25 μm thickness sections on glass slides, dewaxed in xylene (5 × 4 min), and hydrated (100%, 95% and 75% ethanol) for 2×3 minutes each. Slides were heated in a microwave for 3×5 min in target antigen retrieval solution (Dako), cooled down to room temperature, washed in PBS (3x5 min), and blocked and permeabilized in 10% donkey serum containing 0.% triton X-100 for 6 h at 4°C. Slides were incubated in blocking buffer with a primary antibody overnight, and washed with PBS (3 × 5 min) before incubating in blocking buffer for two hours with secondary antibodies (Alexa Fluor 488 or Alexa Fluor 594) (Molecular Probes). Slides were viewed with a fluorescence microscope (Nikon TE2000, Japan).

#### Mice cold endurance experiment

Mice were left in their cage without any padding, food and water in a 4°C cold room. Rectal temperatures were detected by using an electro-thermometer (TH212, Hongauchengyun Co. Ltd., China) every three hours.

#### Statistics

Data were presented as means ± standard deviation (SD). Statistics analysis was performed using SPSS 18.0; multiple group comparisons were made by ANOVA, and two group comparison was determined using unpaired 2-tailed Student’s t test. *P* < 0.05 was considered statistically significant.

## Results

### Electro-acupuncture significantly reduced the ratio of the weight of WAT to body weight

**Figure 1:**
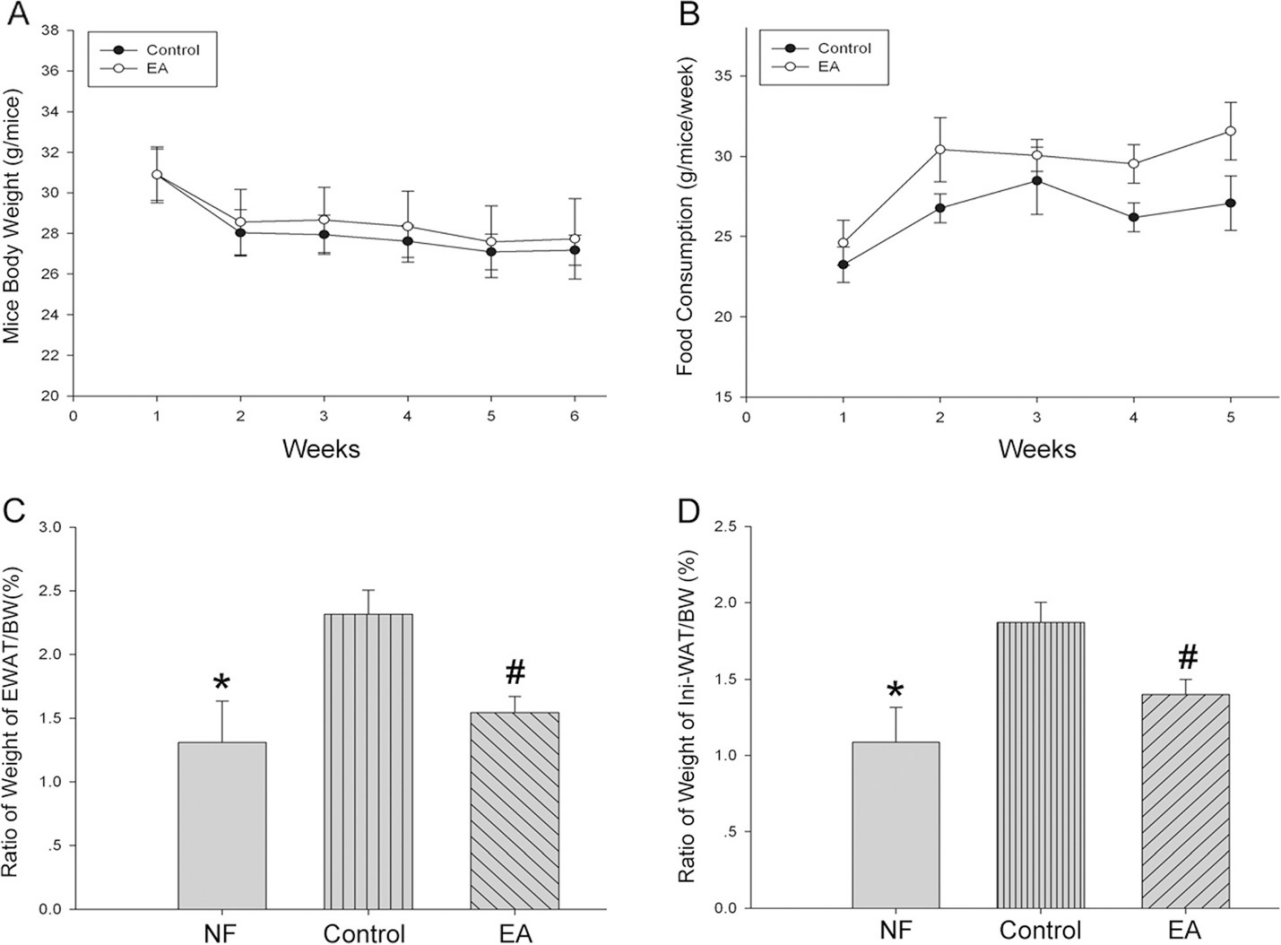
**Effects of acupuncture treatment on diet induced obesity mice (DIO). (A)** Observation of body weight (EA group), n = 28 in each group. **(B)** Measurement of food consumptions n = 28 in each group. **(C)**&**(D)** Ratio of epididymis white adipose tissue weight or inguinal white adipose tissue weight to mouse body weight. n = 20 for NF group, n = 28 for Control and EA group. Data were expressed as means ± SD, * *P* < 0.05 vs Control, # *P* < 0.01 vs Control.

Acupuncture has been used for weight control both clinically and experimentally for many years in China and in other countries [10–13]. However, in this study, electro-acupuncture on Zusanli (ST36) and Neiting (ST44) did not markedly reduce the body weight or the food intake of DIO mice compared with the mice without acupuncture treatment (Figure 1A, 1B), even after five weeks of intervention. For the first three weeks of treatment, mice in both EA and control groups showed a decrease in appetite, which, however, began to decreased by the fifth week of treatment in control mice but continued to increase in EA mice, suggesting that electro-acupuncture enhanced appetite in these mice to a certain extent.

The above observations raised a question: how did electro-acupuncture affect obese mice without significantly reducing body weight or food intake? We isolated mice-epididymal white adipose tissue and inguinal white adipose tissue from the mice in the NF, control, and EA groups, and calculated the ratio of Epi-WAT and Ini-WAT to their body weight. We found that in mice fed a high fat diet, the ratio was significantly higher than that in mice fed normal food. In addition, five weeks of treatment with electro-acupuncture significantly decreased these ratios (Figure 1C, 1D). Our data suggest that the effectiveness of electro-acupuncture on obesity maybe due to the reduction in the ratio of weight of WAT/body weight.

### Electro-acupuncture induced UCP1 expression promoted WAT browning

**Figure 2:**
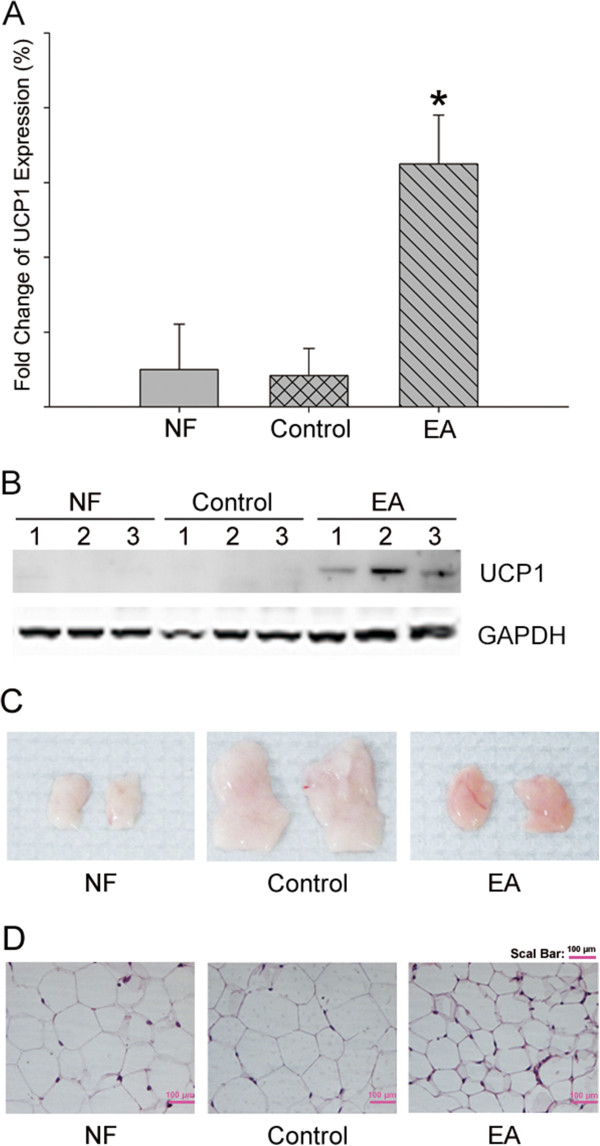
**Electro-acupuncture promoted white adipose tissue browning through induction of UCP1 expression in DIO mice. (A)** mRNA of UCP1 expression in Epi-WAR was detected by qPCR. Data were expressed as means ± SD, n = 16 in each group, * *P* < 0.05 vs Control. Experiment was repeated 3 times independently. **(B)** UCP1 protein expression, n = 3 in each group. Experiment was repeated 3 times independently. **(C)** Representative pictures of Epi-WAT in EA group. **(D)** H&E staining of Epi-WAT in mice EA group and control group.

Un-coupling protein 1 (UCP1) gene is usually specifically expressed in brown adipose tissue (BAT) and is regarded as a BAT marker. In this study, after a five-week treatment with electro-acupuncture, the WAT in EA mice looked grossly darker compared to that in the untreated control mice (Figure 2C), which hinted that electro-acupuncture may promoted WAT to change to BAT. We also found that UCP1 gene expression in WAT increased almost 14 folds in EA mice than in the control mice (Figure 2A), and the UCP1 protein expression also significantly increased in EA mice (Figure 2B). Our data strongly suggests that electro-acupuncture treatment results in browning of WAT in EA mice. Because UCP1 is mainly responsible for thermogenic actions and lipid mobilization in mammals, we analyzed adipocyte size via immunohistochemistry and observed obviously smaller size of the WAT in EA mice compared with the control mice and normal food mice (Figure 2D), suggesting that electro-acupuncture treatment might promote lipolysis in obesity mice.

### Electro-acupuncture treatment increased the ability of cold endurance

**Figure 3:**
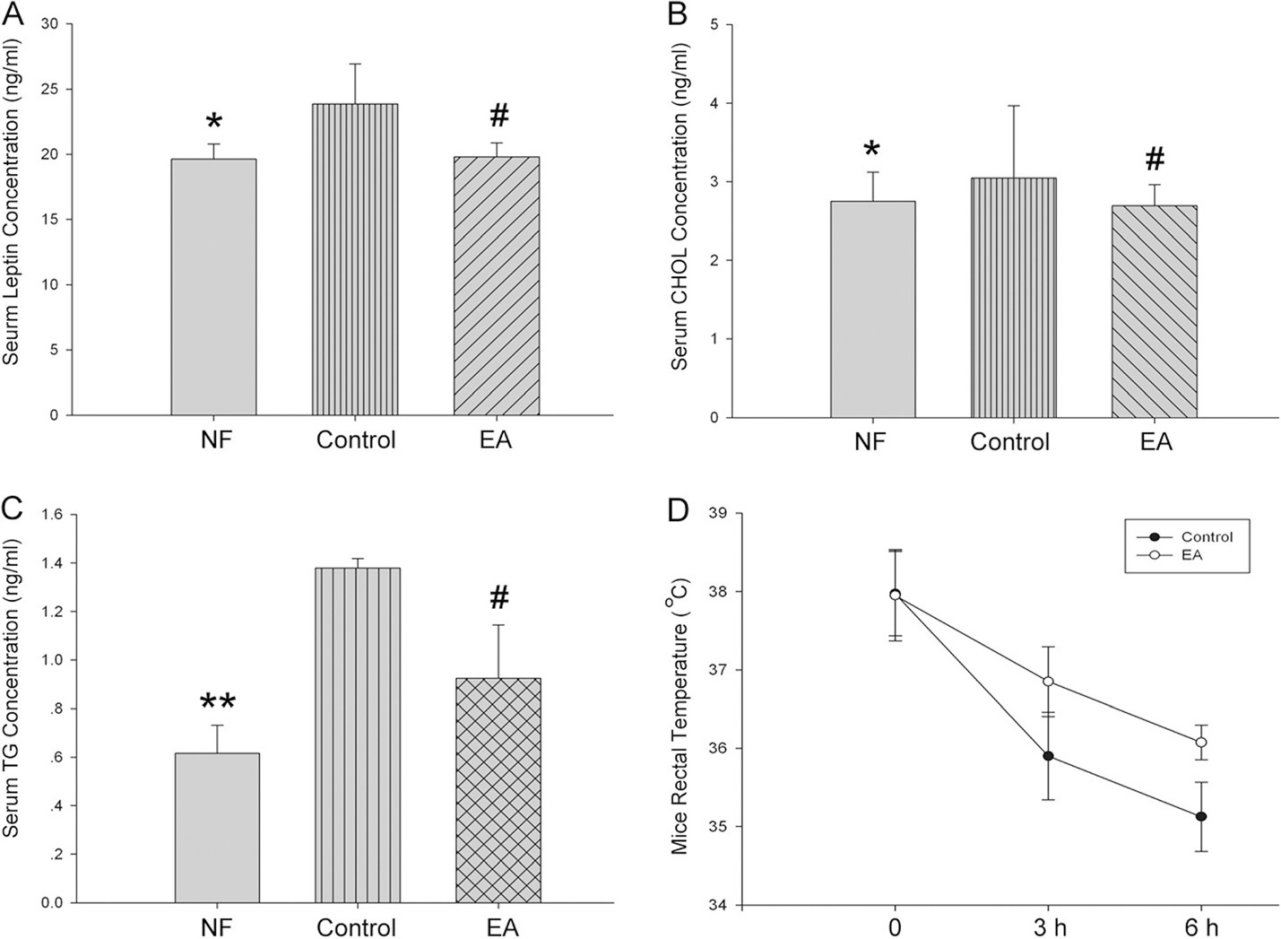
**Electro-acupuncture treatment significantly reversed serum leptin, cholesterol, and triglyceride level in obese mice. (A)** Mouse serum leptin level was detected by ELISA. n = 5 per group. Serum cholesterol **(B)** and TG level **(C)** were measured by ELISA n = 5 in each group, *P < 0.05 vs Control, #*P* < 0.01 vs Control. **(D)** Mice were settled in 4°C cold room, rectal temperature were detected after 3 hours and 6 hours. n = 4 in each group, *P < 0.05 vs Control. All data were expressed as means ± SD, and each experiment was repeated 3 times independently.

To further examine the thermogenesis of mice, four mice from each group were subjected to cold endurance experiment. After exposed to 4°C for six hours, the rectal temperatures of mice that received electro-acupuncture treatment were markedly higher than that of the control mice (Figure 3D), suggesting that EA mice were more able to bear the cold stimulation, compared with the control mice. Combined with the UCP1 expression data, our results suggested that the browning of WAT in the EA mice might be responsible for the generation of more heat to maintain body temperature.

### Electro-acupuncture treatment significantly reversed serum leptin, cholesterol, and triglyceride level of obesity mice

Leptin production can be spontaneously increased by adipocyte in response to excess body fat. Cholesterol and triglyceride also increase in serum when there is abundant lipid in the tissues. Serum level of leptin, cholesterol, and TG were detected by ELISA kit, and we found that compared with mice that were fed normal food leptin, cholesterol, and TG level were all significantly elevated in obese mice after consuming high fat diet for eight weeks; however, five weeks of treatment with electro-acupuncture completely reversed these levels to normal, just as in the NF mice (Figure 3A, 3B and 3C). Our data suggested that electro-acupuncture effectively reduced the lipid content in obese mice.

### Electro-acupuncture treatment changed BAT-related gene expression and Pparγ acetylation in WAT

**Figure 4:**
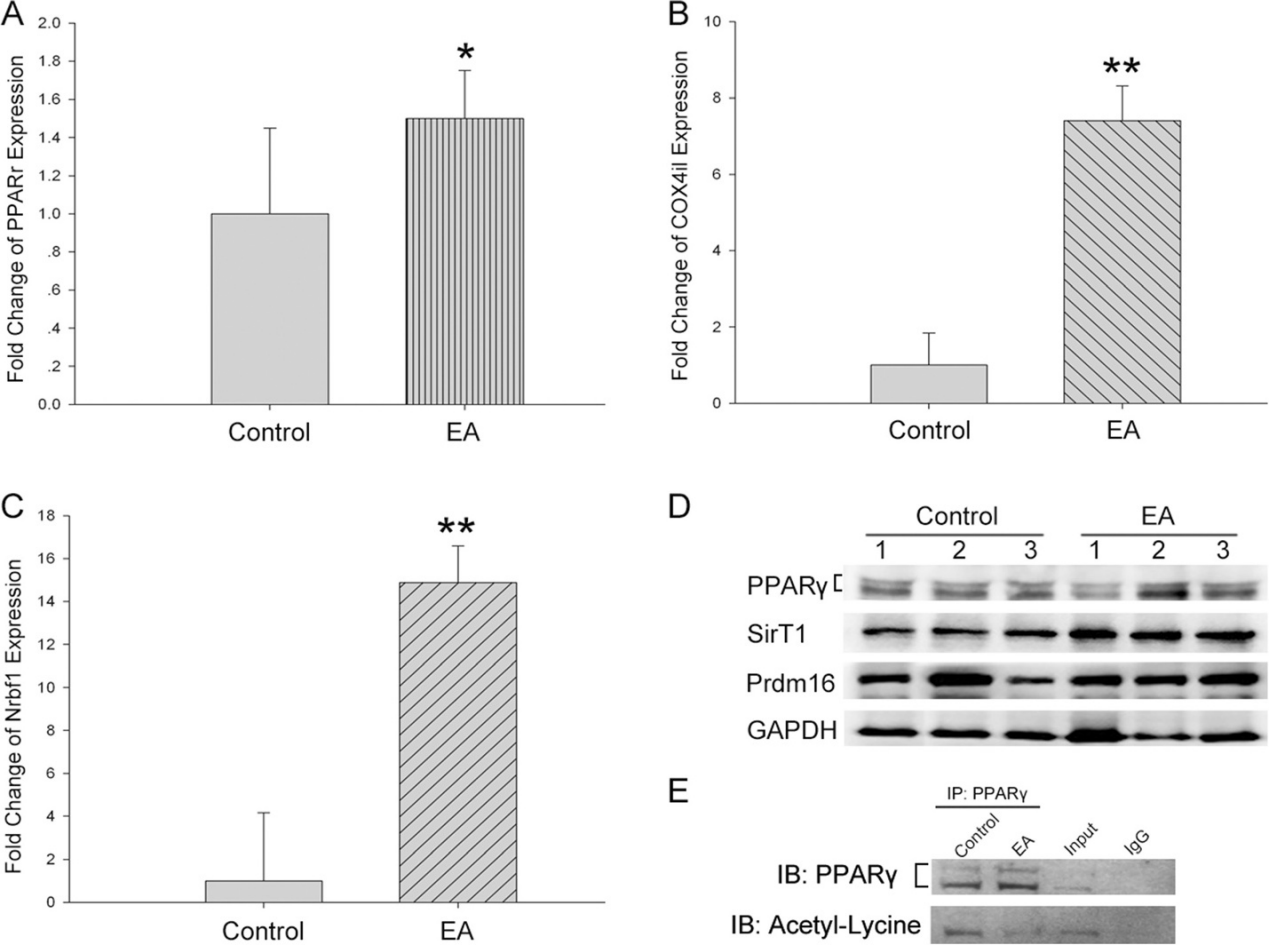
**Electro-acupuncture treatment changed BAT-related gene expression and Pparγ acetylation in WAT.(A)**, COX4il **(B)**, or Nrbf1 **(C)** gene expression in Epi-WAT was measured by qPCR, Data were expressed as means ± SD, *P < 0.05 vs control, **P < 0.01 vs control, n = 12 of each group. **(D)** Immunobloting for protein expressions of Pparγ, SirT1, and Prdm16 in mice epididymis white adipose tissue, n = 3 in each group. Experiment was repeated 3 times independently. **(E)** Acetylation of Pparγ in mice white adipose tissue. Experiment was repeated 3 times independently.

Transcription of UCP1 is regulated by a series of molecular mechanisms, in which Pparγ, SirT1, and Prdm16 are involved. We found that the gene expression of Pparγ increased 1.5 fold in WAT of EA mice than in the control mice (Figure 4A), though its protein level did not change (Figure 4D), but the acetylation of Pparγ was obviously decreased in EA mice after electro-acupuncture treatment (Figure 4E). Prdm16, which is a co-activator of Pparγ regulating UCP1 transcription, did not show any difference in expression levels between the EA and the control groups (Figure 4D). SirT1, which encodes Sirt1 protein and regulates UCP1 expression through histone deacetylation [7], was elevated at the protein level in the EA group (Figure 4D). Unsurprisingly, the gene expressions of other two specific BAT markers, COX4il (Figure 4B) and Nrbf1 (Figure 4C), increased significantly in WAT of EA mice, compared with that of the control mice. These data confirmed, once again, that electro-acupuncture promoted WAT browning.

## Discussion

Obesity is one of the leading global health risk factors, since 1.1 billion people worldwide (>10% of the world population) are classified as overweight [14–16]. Current conventional therapeutic strategies for obesity, including caloric restriction, physical exercise, and drugs, however, can’t effectively achieve adequate weight control. Although exercise can result in short-term weight loss, only 5-10% of subjects can maintain the weight loss for more than a few years [17], and the use of drugs such as Sibutramine, Rimonabant and Orlistat is limited in obesity treatments due to their side effects on the cardiovascular system. Acupuncture is among the oldest healing practices in the world [18]. It exerts its effect through the insertion of thin metallic needles at specific points on the body, and is one of the most rapidly growing complementary therapies. Even in animal models such as obese rats, electro-acupuncture can reduced their body weight [13, 19]. However, our results don’t show a significant reduction in body weight after acupuncture treatment in DIO mice (Figure 1A). A decrease in appetite as a result of acupuncture has been thought as one of the reasons for weight loss in obese mice [13, 20], however on the contrary, we don’t see a significant difference in food intake between the mice with and without electro-acupuncture treatment, and even find a slightly increased food consumption in the EA mice (Figure 1B). Interestingly, the ratio of the weight of WAT, which is a major tissue for excess energy storage, to body weight significant decreases following electro-acupuncture, both for Epi-WAT (Figure 1C) and Ini-WAT (Figure 1D). We suspect that acupuncture treatment promotes WAT lipolysis and restricts adipose tissue synthesis. Thus, this maybe a possible mechanism by which acupuncture can be used to target obesity.

An important characteristic of WAT browning is the over-expression of UCP1, which is seen specifically in BAT. Brown adipocytes uncouple mitochondrial electron transport from ATP synthesis to a greater extent than other cells do by permeabilizing the inner mitochondrial membrane to allow inter-membrane proton to leak back into the mitochondrial matrix, primarily through uncoupling protein-1 (Ucp1) [3] to dissipates energy as heat (nonshivering thermogenesis). Browning of WAT can be brought about by hormones, cytokines, and transcriptional modulation [21–23]. Our results show that five weeks of treatment with electro-acupuncture on Zusanli (ST36) and Neiting (ST44) acupoints can remodel WAT to BAT in obese mice through inducing UCP1 expression (Figure 2A, 2C), thus promoting WAT lipolysis and decreasing adipocyte size as shown in Figure 2D. Since UCP1 is responsible for energy dissipation via nonshivering thermogenesis, under cold circumstances mice with more UCP1 expression can more efficiently generate heat to maintain body temperature as shown in Figure 3D. Also, with the WAT converting to BAT, serum level of leptin is significantly decreased (Figure 3A), as well as TG and cholesterol levels (Figure 3B, 3C). Our experiment, for the first time, provides the evidence of WAT browning by electro-acupuncture on obese mice.

It has recently been reported that activation of the nuclear receptor Pparγ by TZDs such as Rosiglitazone could also induce a brown-like phenotype in white adipocytes through promoting expression of brown adipocyte specific genes (brown genes) and suppressing visceral WAT genes (white genes) [24]. UCP1 expression is regulated by Pparγ binding on UCP1 enhancer. SirT1-dependent deacetylation of Lys268 and Lys293 is required to recruit the BAT program co-activator Prdm16 to Pparγ, leading to selective induction of BAT genes and repression of visceral WAT genes associated with insulin resistance [7]. These findings are consistent with our experiment; in addition WAT browning, expression of Pparγ gene also significantly increase in WAT due to electro-acupuncture treatment (Figure 4A), but not in protein level (Figure 4D). Furthermore, we found the acetylation of Pparγ was obviously decreased in EA mice by the electro-acupuncture treatment (Figure 4E), this data suggest acupuncture might promote Pparγ deacetylation to increase UCP1 gene expression then inducing WAT browning. Moreover, other BAT markers such as Nrbf1 and Cox4il, are also notably elevated by electro-acupuncture (Figure 4B, 4C). Although, we found the electro-acupuncture could promote WAT browning and weight loss in obese mice, the detailed mechanisms by which the effectiveness exerted at the cellular level stay unclear. Further studies in the animal models and clinical patients are of interest.

## Conclusions

Taking together, our results firstly gave the experimental evidence that EA can remodel WAT to BAT through inducing UCP1 expression, and this may be one of the mechanisms by which acupuncture affects weight loss.

## Abbreviations

BAT: Brown adipose tissue
DIO: Diet-induced obesity
EA: Electro-acupuncture
UCP1: Uncouplingprotein-1
WAT: White adipose tissue.
